# 2815. Outcomes of Novel Antimicrobials Compared to Polymyxin B for treatment of infections due Carbapenem-Resistant Enterobacterales Infections

**DOI:** 10.1093/ofid/ofad500.2426

**Published:** 2023-11-27

**Authors:** Tina Zheng, Christine J Kubin, Brian Nelson

**Affiliations:** Maimonides Medical Center , Queens, New York; NewYork-Presbyterian Hospital, New York, New York; NewYork-Presbyterian Hospital, New York, New York

## Abstract

**Background:**

Carbapenem-resistant Gram-negative infections remain a serious threat due to their increasing incidence and high mortality rates. Since 2015, various antimicrobials with in vitro activity against carbapenem-resistant Enterobacterales (CRE) have been approved for use. There is limited evidence on the safety and efficacy of these novel antimicrobials compared to previous first-line agents, polymyxins, for treating CRE infections.

**Methods:**

This was a retrospective cohort study that evaluated the clinical efficacy and safety of the novel antimicrobials compared to polymyxin B. Adult patients with a CRE infection treated with ceftazidime-avibactam (CZA), meropenem-vaborbactam (MVB), or polymyxin B (PMB) for at least 2 days between June 2013 to November 2022 were included. Patients receiving the novel agents (CZA+MVB) were compared to PMB. The primary outcome was 30-day mortality. Secondary outcomes included clinical and microbiological success, 90-day recurrence, development of resistance, frequency of acute kidney injury (AKI), and global success. Global success encompassed 30-day mortality, clinical and microbiological success, and 90-day recurrence.

**Results:**

110 patients were included: 75 in the novel agents group (53 CZA, 22 MVB) and 35 in the PMB group. The median age was 59 years (IQR 45,70), simplified Pitt bacteremia score (qPITT) was 1 (IQR 1,2), and Charlson Comorbidity score was 4 (IQR 2,6). The most common organism was K. pneumoniae (62%) and the most common site of infection was respiratory (54%) followed by blood (22%). 30-day mortality did not differ between the novel agents compared to PMB (23% vs. 29%; p=0.665). Numerically higher clinical success occurred with the novel agents (72% vs. 54%; p=0.106) while microbiological success was similar (93% vs 91%; p=1.0). In the 18 patients who had follow up positive cultures, 3/9 (33%) developed resistance in the novel agents group compared to 7/9 (78%) in the PMB group. No differences were identified in 90-day recurrence and global success. Patients who received novel agents had less AKI compared to the novel agents (16% vs 54%; p< 0.001).

**Conclusion:**

Patients receiving CZA and MVB for CRE infections did not have differences in clinical and microbiological outcomes but had less AKI compared to patients receiving PMB.

**Disclosures:**

**All Authors**: No reported disclosures

